# Case Report: Concurrence of Dermatomyositis and Autoimmune Blistering Diseases: Two Case Reports and a Literature Review

**DOI:** 10.3389/fimmu.2022.855408

**Published:** 2022-03-24

**Authors:** Haixi Wu, Licheng Diao, Ke Xue, Qian Zhao, Xiaoqing Zhao, Qunli Xia, Jie Zheng, Meng Pan, Hua Cao

**Affiliations:** Department of Dermatology, Ruijin Hospital, School of Medicine, Shanghai Jiao Tong University, Shanghai, China

**Keywords:** dermatomyositis, clinically amyopathic dermatomyositis, bullous dermatomyositis, autoimmune blistering disease, malignancy, interstitial lung disease

## Abstract

Dermatomyositis (DM) is an idiopathic inflammatory myopathy primarily involving skin and muscles. Clinically amyopathic dermatomyositis (CADM), a subset of DM, presents with characteristic cutaneous manifestations without clinical evidence of myositis. Although rare, vesiculobullous eruptions could develop in DM patients. Such “bullous DM” is commonly considered a sign of internal malignancy. However, some cases with similar presentations were diagnosed as autoimmune blistering disease eventually. Herein, we reported two cases of CADM with autoimmune blisters formed. Case 1 presented with vesicles and was diagnosed with CADM initially. However, this patient developed blisters again years later and was diagnosed with “pemphigus foliaceous” (PF) accordingly. Case 2, with a history of nasopharyngeal carcinoma and CADM, developed bullous pemphigoid several days after using a heat patch on her abdomen. The association between disease occurrence and local skin damage might provide more evidence to support the “epitope spreading” hypothesis. Moreover, we reviewed related literature and discussed the differences between the two disease entities in clinical presentations, pathogenesis, therapy, and the risk of complications.

## Introduction

Dermatomyositis (DM) is an idiopathic inflammatory myopathy characterized by typical skin lesions and skeletal muscle involvement. Clinically amyopathic dermatomyositis (CADM) is a subset of DM with characteristic cutaneous manifestations and no clinical evidence of myositis (1). These characteristic skin lesions include heliotrope, Gottron’s papules, and Gottron’s sign. However, vesiculobullous eruptions are rarely observed. If blisters are presented, they are defined as “bullous DM” and are usually considered a sign of internal malignancy (2).

Nevertheless, it was also noted that some mimics can be diagnosed as autoimmune blistering diseases (AIBDs). AIBDs involve a group of dermatoses that autoantibodies bind to antigens in the skin and mucous membranes. AIBDs can be divided into three types, subepidermal split formation (pemphigoid diseases), acantholysis (pemphigus), and dermatitis herpetiformis. Furthermore, the common pemphigoid diseases include bullous pemphigoid (BP), mucous membrane pemphigoid (MMP), and epidermolysis bullosa acquisita (EBA). In contrast, the common pemphigus includes pemphigus vulgaris (PV) and pemphigus foliaceous (PF).

Herein, we reported two cases of CADM with blisters. Case 1 presented with characteristic DM skin lesions and vesicles on the lower back, without muscle weakness or tenderness. The biopsies from erythema and vesicle showed compatible with DM and severe edema of the dermal papilla, respectively. Therefore, the diagnosis of “bullous CADM” was made. Years later, the patient developed blisters and erosions again. This time, the histology, immunohistopathology, and serology tests all suggested the diagnosis of “PF”. Case 2, with a medical history of nasopharyngeal carcinoma (NPC) and CADM, this patient developed erythema and dense blisters several days after using a heat patch. “BP” was made with the evidence of histology and serologic findings. These two cases presented with a unique disease progress with different patterns of blister formation and skin damage-triggered AIBD, respectively.

## Case Description

### Case 1

**Figure 1:**
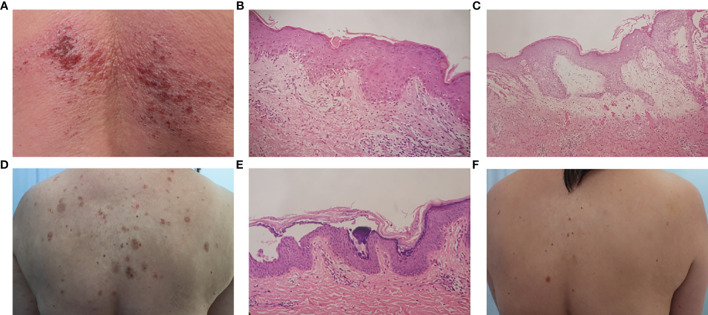
**(A)** Erythema and vesicles formation on the lower back. **(B)** Histology from an erythema on the upper back showed focal parakeratosis of the epidermis, liquefaction of basal cells, edema of dermal papilla, and mild superficial and middle perivascular lymphocyte infiltration. **(C)** Histology from a vesicle on the lower back showed severe edema of dermal papilla and perivascular lymphocyte infiltration. **(D)** Multiple ruptured bullae and erosions on the back. **(E)** Histological examination from a bulla on the abdomen showed acantholysis and intraepidermal bulla formation. **(F)** The patient’s condition was stable at the last follow-up.

In 2017, a 55-year-old Chinese woman presented at the study hospital with 2-month history of skin erythema and 1 week of vesicles. Physical examination showed periorbital erythema, Gottron’s papules, Gottron’s sign, and erythema on the upper chest and back. Clustered dense vesicles were also observed on her lower back (**Figure 1A**). She had no muscle weakness or muscle tenderness. This patient had a medical history of hypertension and took telmisartan and felodipine for 2 years. Laboratory investigations showed increased levels of lactate dehydrogenase (LDH) [247 IU/L, normal range (NR) 98–192] and aspartate aminotransferase (AST) (50 IU/L, NR 8–40). Myositis-specific antibodies (MSAs) were also positive, including anti-small ubiquitin-like modifier activating enzyme (SAE) and -Jo-1 antibodies (immunoblotting). However, her creatine kinase (CK) was normal (92 IU/L, NR 22–269). No significant abnormality was found on electromyogram (EMG), muscle magnetic resonance imaging (MRI), or muscle biopsy. One skin biopsy from an erythema lesion on the back showed liquefaction of basal cells, edema of the dermal papilla, and mild superficial and middle perivascular lymphocyte infiltration (**Figure 1B**). Another skin biopsy from a vesicle lesion showed severe edema of the dermal papilla and perivascular lymphocyte infiltration (**Figure 1C**). Direct immunofluorescence (DIF) was negative. Screening for interstitial lung disease (ILD) and malignancy was negative. The patient was diagnosed with bullous CADM and treated with methylprednisolone 40 mg/day and hydroxychloroquine 200 mg/day initially. The dose of methylprednisolone was tapered after 2 weeks of treatment due to the significantly reduced skin eruptions and decreased levels of LDH and AST (201 and 16 IU/L, respectively).

Approximately one and a half years later, the patient was hospitalized because of itchy blisters over a month and the dose of prednisone that time was 12.5 mg/day. Physical examination showed some ruptured bullae and erosions on the trunk and extremities (**Figure 1D**). Immunological findings in serum by ELISA showed positive anti-desmoglein (Dsg) 1 antibody (130.5 U/ml, NR ≤20) and negative anti-Dsg3 antibody. Histopathological examination showed acantholysis and intraepidermal bulla formation (**Figure 1E**). DIF revealed intercellular binding of IgG and C3 within the epidermis. Repeat screening for ILD and malignancy was negative. Based on these results, CADM associated with PF was diagnosed. The patient was treated with oral prednisone 12.5 mg/day, methotrexate 7.5 mg/week, and topical halometasone cream. After 2 months, erosions were controlled and the prednisone dose was tapered. At the last follow-up in December 2021, the skin condition was stable with very few skin eruptions (**Figure 1F**).

### Case 2

In 2017, a 68-year-old Chinese woman presented a 1-year history of purple erythema on her face. Physical examination showed periorbital and malar purple erythema without muscle weakness or tenderness. She took 2 months of radiotherapy in 2015 due to NPC. Laboratory investigations showed normal levels of muscle enzymes. MSAs showed anti-transcription intermediary factor 1-γ (TIF1-γ) antibody positive (immunoblotting, ELISA), and skin biopsy showed focal liquefaction and degeneration of basal cells, tortuosity of small vessels, and mild perivascular lymphocyte infiltration. Screening for ILD and internal malignancy was negative. The patient was diagnosed with CADM and treated with thalidomide 50 mg/day and hydroxychloroquine 200 mg/day.

**Figure 2:**
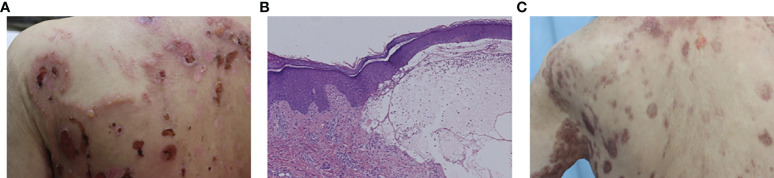
**(A)** Bullae and erosions presented on the back. **(B)** Histology from a bulla on the right forearm showed subepidermal bulla formation and the bullous fluid and the superficial dermis were infiltrated by eosinophils. **(C)** The bullous eruptions were stable 2 months later.

This patient developed abdominal bullous eruptions 2 years later after using a heat patch for several days. The eruptions got aggravated and widespread to the whole body within a week. Physical examination showed widespread erythema with bullae and erosions on the trunk, neck, and extremities, including the armpits and groins (**Figure 2A**). The Nikolsky’s sign was negative. Immunological findings showed positive anti-BP180 antibody (138.16 U/ml, NR ≤9), while anti-Dsg1, -Dsg3, and -BP230 antibodies were negative. Histology showed a subepidermal bulla and eosinophil infiltration in the bullous fluid and the superficial dermis (**Figure 2B**). Both DIF and indirect immunofluorescence (IIF) were negative. The patient was diagnosed with CADM associated with BP and treated with oral hydroxychloroquine 200 mg/day, minocycline 200 mg/day, and topical halometasone cream. Two months later, the bullous eruptions were controlled (**Figure 2C**). During our latest follow-up in September 2021, this patient presented with no sign of recurrence of BP or malignancy.

## Discussion

DM with blister has been reported in different terms in the literature, including DM with vesicle formation, vesiculobullous DM, and bullous DM (3). Apart from this, as reported in this study, there were a total of 14 cases reported so far in the literature that DM patients with blisters turned out to be AIBD eventually (**Table 1**) (4–16).

**Table 1:** A summary of reported cases of DM associated with AIBDs.

	Age	Sex	DM subset	AIBD	Time from onset of DM to AIBD^a^	Possible Trigger	MSAs	ILD	Malignancy
White and Tesar (4)	67	Female	DM	DH	3 years	(-)	/	(-)	(-)
Kalovidouris et al. (5) Case 1^b^	34	Male	DM	DH	-7 years	(-)	Anti-Jo-1 Ab	(-)	(-)
Barrows-Wade and Jordon (6)	56	Female	DM	Linear IgA bullous dermatosis	4 months	(-)	(-)	(-)	(-)
Glover and Leigh (7)	65	Male	DM	BP	Simultaneously	(-)	/	(-)	(-)
Tsukada et al. (8)	70	Male	DM	BP	8 months	(-)	/	(-)	(-)
Narbutt et al. (9)	11	Male	Juvenile DM	PF	2 years	Enalapril	/	(-)	(-)
Yanagi et al. (10)	81	Female	DM	BP	3 months	Surgery	/	(-)	Colon carcinoma
Ahmad and Nabih (11)	53	Female	DM	PF	15 years	Lisinopril	/	(-)	(-)
Black and Marshman (12)	76	Female	DM	PV	3.5 years	(-)	/	(-)	(-)
Thongprasom et al. (13)	36	Female	CADM	PV	-4 years	(-)	/	(-)	(-)
Fujimoto et al. (14)	39	Female	CADM	PF	2 years	(-)	Anti-NXP2 Ab	(-)	(-)
Garcia et al. (15)	69	Female	CADM	BP	Simultaneously	(-)	Anti-MDA5 Ab	(+)	MALT gastric lymphoma
Patsatsi et al. (16) Case 1	51	Female	DM	PV	-8 months	(-)	/	(-)	(-)
Case 2	82	Male	CADM	MMP	-14 years	(-)	/	(+)	(-)
Present Case 1	55	Female	CADM	PF	1.5 years	(-)	Anti-SAE, -Jo-1 Ab	(-)	(-)
Present Case 2	68	Female	CADM	BP	3 years	Heat patch	Anti-TIF1-γ Ab	(-)	NPC

DM, dermatomyositis; AIBDs, autoimmune blistering diseases; MSA, myositis-specific antibody; CADM, clinically amyopathic dermatomyositis; ILD, interstitial lung disease; DH, dermatitis herpetiformis; PV, pemphigus vulgaris; PF, pemphigus foliaceous; BP, bullous pemphigoid; MMP, mucous membrane pemphigoid; NPC, nasopharyngeal carcinoma; MALT, mucosa-associated lymphatic tissue; NXP2, nuclear matrix protein 2; MDA5, melanoma differentiation-associated gene 5; SAE, small ubiquitin-like modifier activating enzyme; TIF1, transcription intermediary factor 1.

a Time span from the onset of first symptom assumed to be related to DM to AIBD, the symbol “-” means AIBD occurred before DM.

b Case 2 in this literature was polymyositis.

Fujimoto et al. (14) reported a 39-year-old Japanese woman who presented with PF over 2 years after the onset of CADM. MSAs of that patient showed anti-nuclear matrix protein 2 antibody positive, which is a risk factor of malignancy. Our Case 1 presented a similar disease process with different positive MSAs (i.e., anti- SAE and -Jo-1 antibodies). Additionally, to the best of our knowledge, Case 1 was the first report of vesiculobullous eruptions formed in autoimmune disease at different clinical stages with distinct mechanisms, suggesting that blisters in DM patients could be caused by the aggravation of the primary disease or other autoimmune diseases. Garcia et al. (15) reported a 69-year-old woman who was diagnosed with BP and CADM simultaneously when treated for mucosa-associated lymphoid tissue (MALT) gastric lymphoma. This woman also showed positive anti-melanoma differentiation-associated gene 5 antibody, which is a risk factor of rapid progressive ILD (15). Differently, our Case 2, with a 3-year history of CADM and a 4-year history of NPC, presented with positive anti-TIF1-γ antibody, a biomarker associated with malignancy. Furthermore, Case 2 showed typical progress of BP after using a heat patch, which might imply that the exposure of an antigen caused by local cutaneous damage played an essential role in the pathogenesis.

Though both disease entities, bullous DM and AIBD, manifested as blister formation in DM patients, they had respective features to recognize. Moreover, due to the dissimilar pathogenesis, the differences in therapy and risk of complications should be considered. Clinically, blisters in bullous DM patients commonly present an erythema background with negative Nikolsky’s sign. Such blisters tend to appear on irritated areas such as the extensors of the arms, knees, upper chest, and back (2). The period of its occurrence is transient and in which there is usually a concurrence of edematous erythema elsewhere. Histopathology of bullous DM commonly presents with papillary dermal edema and mucin deposition with negative DIF. No specific antibodies are detected. However, the vesiculobullous eruptions of AIBDs have unique features of skin manifestation, histopathology, immunopathology, and serum immunology. Pathogenetically, the parallel of bullae and DM was observed (17). It was even postulated that the two clinical forms, edematous and vesiculobullous, could be the same or overlapping manifestations (18). Contrarily, while the AIBD erupted, both of our patients showed no evidence of aggravation of CADM. The association between DM and AIBD remains unknown. AIBD can occur before, simultaneously with, or after the onset of DM. The most acceptable hypothesis for the coexistence of these two autoimmune diseases is epitope spreading. Sequestered antigens being exposed due to tissue damage can lead to a secondary autoimmune disease in some situations (19). Case 2 developed BP after applying a heat patch to her abdomen, which might directly damage the local skin tissue or lead to contact dermatitis. With exposure to autoantigens, this patient may eventually develop BP. Therapeutically, when Case 1 presented vesicles during her first-time hospital visit, we focused on the acute progress of CADM and treated her with methylprednisolone 40 mg/day. However, when she got readmitted to the hospital, we focused on the eruption of AIBD and the therapy was much more prudent. The skin lesions of both cases got relieved after an additional therapy with topical halometasone and immunosuppressant without increasing the corticosteroid.

The primarily fatal complications of DM are malignancy and ILD. The association between bullous DM and malignancy has been validated. It is reported that the risk of developing malignancy in bullous DM can reach as high as 68% (2). As for DM associated with AIBD, among 16 cases (14 cases reported historically and two cases in this study), three of them (18.8%) had a malignant tumor, including colon carcinoma, MALT gastric lymphoma, and NPC. It was notable that all three cases were BP, which accounted for 60% (3/5) among all BP cases. At present, the association between bullous DM and ILD is still uncertain. Among all cases, two of them were diagnosed with ILD (12.5%), and both were diagnosed with CADM. During our last clinical follow-up, Case 1 presented with no sign of malignancy and Case 2 showed no evidence of new malignancy or NPC recurrence. Our follow-up showed no link between blister formation and malignancy in CADM. Nevertheless, a longer follow-up and more cases are warranted for such validations.

## Data Availability Statement

The raw data supporting the conclusions of this article will be made available by the authors without undue reservation.

## Ethics Statement

Written informed consent was obtained from the individuals for the publication of any potentially identifiable images or data included in this article.

## Author Contributions

HXW: article writing. LCD: serology tests. KX and QZ: patient management. XQZ: histopathology support. QLX: biopsies. JZ, MP, and HC: article revising. All authors contributed to the article and approved the submitted version.

## Funding

This study received funding from the National Natural Science Foundation of China (81573037, 81872523, 82073432), National Key Clinical Specialty (2012649), Science and Technology Commission of Shanghai Municipality (134119a6100), Clinical Research Plan of SHDC (16CR3084B), Shanghai Municipal Education Commission–Gaofeng Clinical Medicine Grant Support (20172009), and Shanghai Yiyuan Rising Star Outstanding Young Medical Talents (2019).

## Conflict of Interest

The authors declare that the research was conducted in the absence of any commercial or financial relationships that could be construed as a potential conflict of interest.

The handling editor ZC declared a shared parent affiliation with the authors at the time of review.

## Publisher’s Note

All claims expressed in this article are solely those of the authors and do not necessarily represent those of their affiliated organizations, or those of the publisher, the editors and the reviewers. Any product that may be evaluated in this article, or claim that may be made by its manufacturer, is not guaranteed or endorsed by the publisher.
